# Early Diagnosis of Ischemic Stroke on Non-Contrast CT Scans Using a Convolutional Neural Network: A Case Study from Mulago National Referral Hospital, Uganda

**DOI:** 10.21203/rs.3.rs-9914433/v1

**Published:** 2026-07-01

**Authors:** Michael Kateregga, Irene Wanyana, Richard Keith Kwagala, James Senoga, Success Kamuhanda, Mike Nsubuga, Charles Batte, Rornald Muhumuza Kananura

**Affiliations:** Makerere University; Makerere University; Johns Hopkins University; Ernest Cook University; Makerere University; University of Bristol; Makerere University; Makerere University

## Abstract

**Background:**

In low- and middle-income countries, the scarcity of specialist radiologists contributes to delays in ischemic stroke diagnosis. Early recognition is essential for initiating interventions that can reduce morbidity and mortality.

**Objective:**

To develop and assess a convolutional neural network (CNN) capable of detecting early ischemic stroke on non-contrast computed tomography (NCCT) scans and to develop a web-based platform for demonstrating potential clinical application.

**Methods:**

A retrospective diagnostic accuracy study was performed using 1,000 NCCT scans (500 stroke positive, 500 controls) from adult patients at Mulago National Referral Hospital between January 2022 and June 2023. Lesions were independently annotated by three radiologists (κ = 0.83). Preprocessing included intensity normalization and texture analysis. The CNN was trained with dropout, L2 regularization, and batch normalization. Five-fold cross-validation was conducted. Performance was compared with a senior radiologist (> 5 years’ experience) using a balanced 30- case subset. A web-based interface was created for deployment.

**Results:**

The CNN achieved a mean accuracy of 90.2% (± 1.8; 95% CI: 88.0–92.4), sensitivity of 91.4% (± 1.7; 95% CI: 89.3–93.5), specificity of 89.0% (± 2.1; 95% CI: 86.4–91.6), and an area under the receiver operating characteristic curve (AUC) of 91.3% (± 1.7; 95% CI: 89.2–93.4) across five-fold cross-validation. On the independent radiologist comparison subset, the CNN achieved a sensitivity of 86.7% and specificity of 88.7%, missing two stroke cases, while the senior radiologist achieved a sensitivity of 100.0% and specificity of 95.0% with no missed stroke cases. Despite the radiologist demonstrating slightly higher diagnostic performance, the overall difference was not statistically significant (p = 0.21). The model processed scans in approximately 3 seconds per case, compared with approximately 5 minutes required for radiologist interpretation. In addition, a browser-based interface was developed to support scan upload and automated analysis for potential clinical deployment.

**Conclusion:**

The model demonstrated accuracy comparable to that of a senior radiologist while delivering results in significantly less time. These findings suggest that the integrated web platform has promising potential to support stroke triage, particularly in settings with limited radiology expertise.

## Introduction

In low- and middle income countries (LMICs), the burden is disproportionately high due to limited access to timely diagnosis, acute care, and preventive strategies(1). In Uganda, the incidence is rising, driven largely by increased prevalence of hypertension, diabetes, and other vascular risk factors(2).

Non-contrast computed tomography (NCCT) is the most widely available imaging modality for suspected stroke in Uganda. While it is essential for excluding hemorrhage, detecting early ischemic changes on NCCT is challenging and often requires experienced interpretation(3). The shortage of radiologists in many public hospitals means that such expertise is not always available in a timely manner, delaying diagnosis and treatment.

Several studies have shown that computer-assisted systems can support clinicians in identifying early ischemic changes(4, 5). However, many existing solutions depend on advanced imaging such as Computed Tomography angiography or perfusion studies, which are not routinely available in low-resource environments. Others have demonstrated promising accuracy but have not been adapted into formats usable at the point of care(6, 7).

The present work sought to design and validate a Convulational Neural Network(CNN) capable of identifying early ischemic stroke on NCCT, trained exclusively on data collected in Uganda. A secondary goal was to create a deployment-ready interface that could integrate into everyday hospital workflows.

## Methodology

### Study Design and Setting

This study employed a retrospective, quantitative design to develop and evaluate a CNN for the early detection of ischemic stroke on NCCT scans. The research was conducted at Mulago National Referral Hospital in Kampala, Uganda the country’s largest public healthcare facility and primary tertiary referral center. Mulago manages a substantial volume of stroke cases annually, with two operational CT scanners and a radiology department staffed by three in-house radiologists and five adjunct radiologists from Makerere University. Despite this capacity, reporting delays of three to five days are common due to prioritization of trauma and other emergencies, highlighting the need for expedited diagnostic tools.

## Data Collection and Preparation

A total of 1,450 non-contrast CT (NCCT) scans were retrieved from the hospital’s radiology archive. After quality control screening to exclude images with motion artifacts, incomplete series, non-brain windowing, or corrupted metadata, 1,000 scans were retained 500 with radiologically confirmed ischemic stroke and 500 controls without stroke. All scans were exported in Digital Imaging and Communications in Medicine (DICOM) format and fully anonymized in compliance with ethical guidelines. The dataset was curated solely based on imaging features, with no patient demographic or clinical data used.

Lesion annotation was performed on the 500 stroke-positive scans by three radiologists with at least five years of neuroimaging experience. The annotation process targeted established early ischemic signs, including hypoattenuation, sulcal effacement, and loss of gray–white matter differentiation. A semi-automated Python-based pipeline incorporating the Roboflow API and OpenCV was used to standardize annotation files and formats. Inter-rater reliability, assessed using Cohen’s kappa coefficient, was 0.83, indicating strong agreement. Discrepancies, present in approximately 6.7% of cases, were resolved by consensus, and the resulting masks were used as the reference standard for model development.

## Feature Extraction and Preprocessing

Image preprocessing included intensity normalization, resizing to a standardized input dimension, and augmentation via rotations, translations, and flips to enhance variability and mitigate overfitting. Quantitative features extracted from annotated lesion areas included intensity metrics (mean, standard deviation), shape descriptors (area, perimeter, circularity), and texture features based on Gray Level Co-occurrence Matrix properties such as contrast, energy, and homogeneity. Class balancing was maintained by stratifying the dataset into equal proportions of stroke-positive and control images.

## Model Architecture and Training

**Figure 1:**
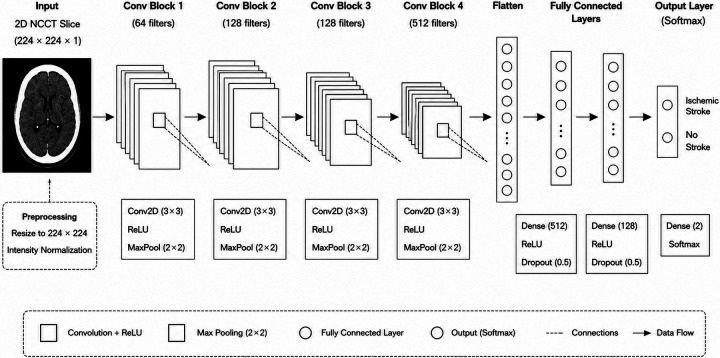
CNN architecture for early ischemic stroke detection using NCCT images.

The model was developed to analyse individual 2D slices extracted from non-contrast CT scans and learn image features associated with early ischemic stroke. Each slice passed through a series of convolution and pooling layers that progressively extracted and condensed relevant information before classification through fully connected layers and a softmax output. The overall architecture of the CNN model is illustrated in Fig. 1. To improve stability and reduce overfitting, dropout and L2 regularization were applied during training. Training was performed using the Adam optimizer over 100 epochs in PyTorch with GPU acceleration. Although image processing occurred at the slice level, diagnostic performance was evaluated at the patient (scan) level by combining slice predictions into a single final classification for each NCCT study.

## Model Evaluation

A five-fold cross-validation strategy was implemented to ensure robust performance assessment. In each fold, the model was trained on 80% of the data and tested on 20%, with each subset serving as the test set once. Evaluation metrics included sensitivity, specificity, accuracy, and AUC, with definitions aligned to established diagnostic performance measures(8). Confusion matrices and calibration curves were generated to further assess classification reliability and probability calibration.

### Comparative Analysis with Radiologist Performance

To determine clinical relevance, the CNN was compared against the diagnostic performance of a senior consultant radiologist. A balanced test set of 30 anonymized NCCT scans (15 ischemic stroke, 15 non-stroke), which was strictly held out from all model training and cross-validation procedures, was independently reviewed by both the model and the radiologist, blinded to clinical history. Sensitivity, specificity, and average interpretation time were recorded. Statistical comparison of the paired binary classification outcomes was performed using McNemar’s test, with significance set at p < .05.

## User Interface Development

To demonstrate clinical applicability, a web-based interface was developed on top of a RESTful API to enable non-technical healthcare staff to interact with the model. The interface supported secure log-in, DICOM upload, automated inference, probability visualization, and downloadable PDF reports. Frontend components were built using HTML, CSS, and JavaScript, while Flask powered the backend. The system was tested in a simulated hospital environment using anonymized scans.

## Ethical Considerations

### Ethical approval

was obtained from the Institutional Review Board of the Makerere University School of Public Health (MakSPH-REC 566). Data use was authorized by Mulago National Referral Hospital administration. A waiver of informed consent was granted by the approving Research Ethics Committee because the study utilized retrospectively collected and anonymized CT scan images. All scans were anonymized to remove identifiable information, stored securely on encrypted drives, and accessed only by authorized personnel. The study adhered to Uganda’s Data Protection and Privacy Act and relevant international guidelines, including the General Data Protection Regulation.

## Results

### Dataset Composition and Annotation

**Figure 2:**
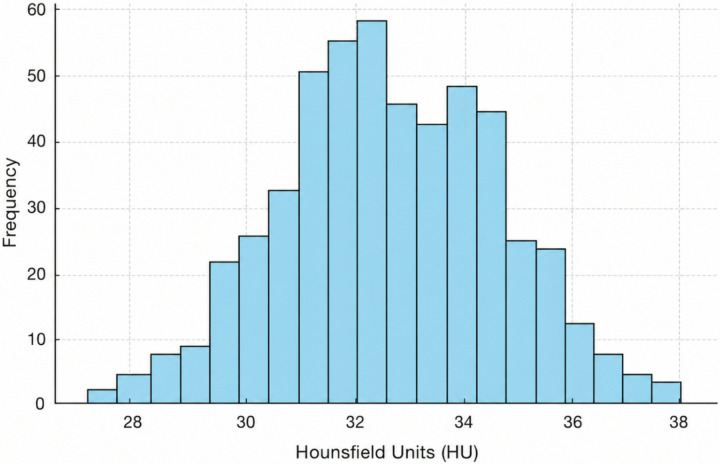
Histogram showing the distribution of Hounsfield Unit values among annotated ischemic stroke lesions on non-contrast CT scans.

Most annotated ischemic lesions demonstrated hypoattenuation within the range of 30–35 Hounsfield Units (HU), consistent with early infarct core presentations. A smaller subset exhibited higher attenuation values, possibly reflecting partial infarcts or overlapping anatomical structures. The distribution of Hounsfield Unit values among annotated ischemic lesions is shown in Fig. 2.

### Preprocessing, Feature Extraction, and Cross-Validation Performance

**Figure 3:**
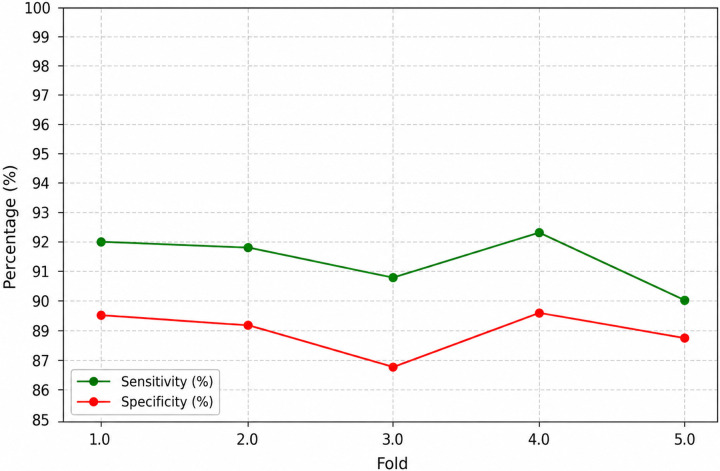
Sensitivity and specificity of the CNN model across the five cross-validation folds.

Following preprocessing, which included intensity normalization, histogram equalization, and texture-based feature enhancement, the model was trained and evaluated using five-fold cross validation. Performance remained relatively consistent across validation folds, demonstrating stable generalization to unseen cases. The CNN achieved a mean sensitivity of 91.4% (± 1.7) and a mean specificity of 89.0% (± 2.1), indicating strong ability to identify ischemic stroke cases while maintaining acceptable discrimination of non-stroke scans. Sensitivity and specificity across the five validation folds are presented in Fig. 3. The observed variability across folds was minimal, suggesting that the model maintained robust performance throughout the cross validation process.

### Overall Model Performance

**Figure 4:**
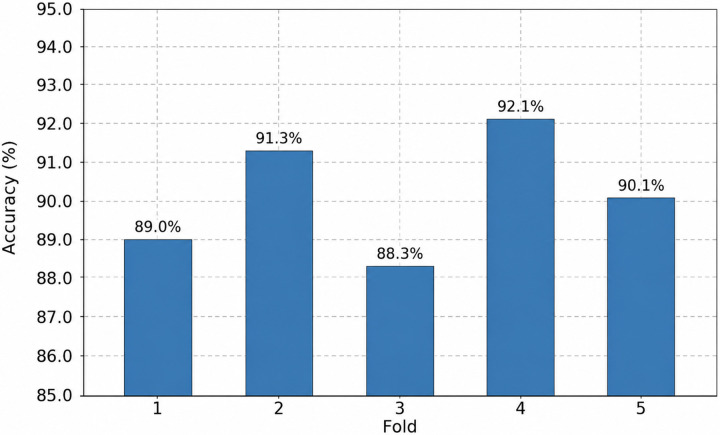
Cross-validation accuracy of the CNN model across the five validation folds.

**Figure 5:**
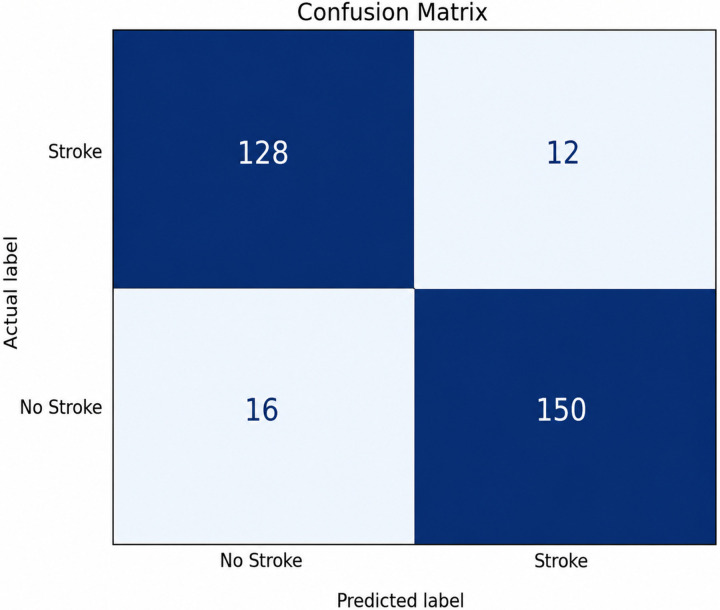
Confusion matrix illustrating the classification performance of the CNN model for ischemic stroke and non-stroke scans.

**Figure 6:**
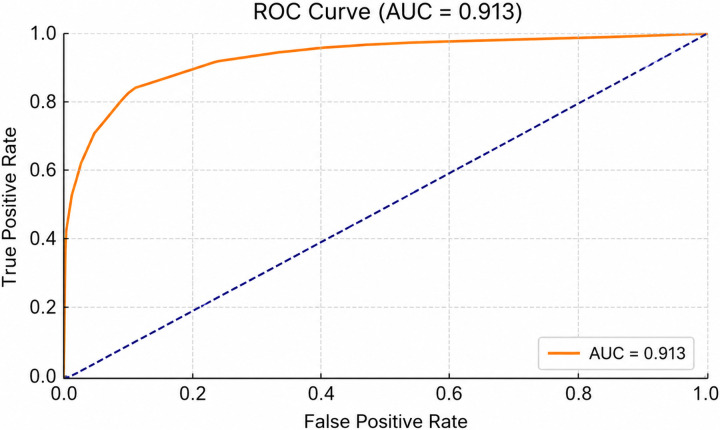
Receiver operating characteristic curve of the CNN model for ischemic stroke detection on the evaluation dataset.

The overall diagnostic performance of the CNN model across the five-fold cross-validation process is summarized in Table 1. The model achieved a mean accuracy of 90.2% (± 1.8), sensitivity of 91.4% (± 1.7), specificity of 89.0% (± 2.1), and an AUC of 91.3% (± 1.7). Accuracy across the five validation folds ranged from 88.3% to 92.1%, demonstrating relatively stable performance throughout the cross-validation process, as shown in Fig. 4. To further evaluate classification performance, a confusion matrix was generated using the pooled evaluation dataset seen in Fig. 5, illustrating the distribution of true-positive, true-negative, false-positive, and false-negative classifications. The model's ability to discriminate between ischemic stroke and non-stroke scans was further assessed using receiver operating characteristic analysis, with the ROC curve presented in Fig. 6 demonstrating an AUC of 0.913, indicating good diagnostic discrimination.

### Radiologist Comparison

The comparison of the CNN with the radiologist summarized in Table 2 demonstrated that the radiologist achieved slightly higher diagnostic performance on the independent evaluation subset. The CNN missed two stroke cases, while the radiologist identified all stroke cases correctly. However, the overall difference in diagnostic accuracy was not statistically significant (Wilcoxon signed-rank test, p = 0.21). Notably, the CNN generated results substantially faster than the radiologist, requiring approximately 3 seconds per scan compared with approximately 5 minutes for manual interpretation, highlighting its potential utility in emergency stroke care.

### Deployment Platform

**Figure 7:**
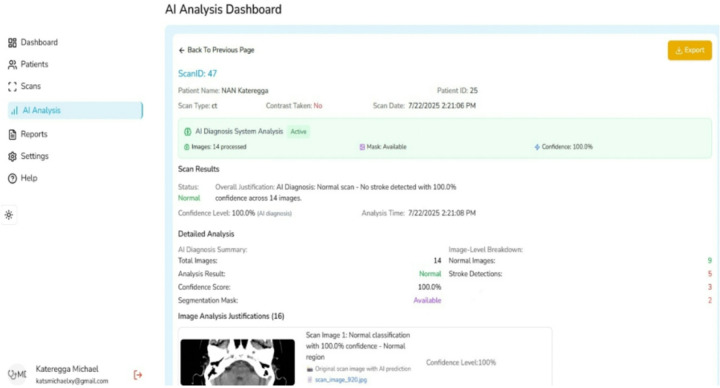
Screenshot of the web-based stroke detection platform showing automated analysis results generated by the CNN model.

A web-based user interface was developed and connected to the model through a RESTful API. The platform begins with a secure login, after which the user can upload a brain scan in DICOM, JPEG, or PNG format. Once the scan is uploaded, the system automatically analyzes the image in the background and, within a few seconds, displays the classification result on the dashboard, indicating whether the scan shows ischemic stroke or is normal. A screenshot of the developed platform and its automated analysis dashboard is presented in Fig. 7.

The diagnostic times reported in Table 2 were measured using this operational platform on the 30- case evaluation set. These results confirm that the system can deliver automated analysis approximately 100 times faster than a senior radiologist interpreting the same scans manually. By relying only on routinely available imaging formats and a standard computer with internet access, the platform directly addresses the delays in stroke diagnosis highlighted in the introduction. It provides clinicians with rapid, reproducible results that can guide early triage decisions even in settings with limited radiology expertise.

While the platform has not yet undergone live clinical deployment, testing in a simulated environment with anonymized data demonstrated full operational functionality and usability for non-technical healthcare staff.

## Discussion

This study demonstrated that a CNN trained on non-contrast CT scans can achieve strong diagnostic performance for early ischemic stroke detection in a low-resource setting. The model achieved a mean accuracy of 90.2%, sensitivity of 91.4%, specificity of 89.0%, and an AUC of 91.3% across five-fold cross-validation. These findings are comparable to previous studies that reported diagnostic accuracies ranging between 87% and 93% for deep learning approaches in ischemic stroke detection(9, 10). Unlike several existing models developed using advanced imaging modalities such as CT perfusion or angiography, the present study relied exclusively on non-contrast CT scans, which are more widely available in Ugandan public hospitals and other low-resource settings.

The diagnostic performance observed in this study was slightly lower than the near-perfect results reported by some highly specialized deep learning systems developed using multicenter datasets and advanced imaging protocols(11). However, the current findings may better reflect realistic clinical performance within routine hospital environments where imaging quality, scanner variability, and resource limitations can affect model accuracy. Importantly, the CNN maintained relatively high sensitivity, indicating good ability to identify most stroke cases while minimizing missed diagnoses. This is clinically relevant because delayed recognition of ischemic stroke can significantly worsen patient outcomes.

Compared with the work by (12) which reported difficulty detecting subtle infarcts within regions such as the basal ganglia and insular cortex, the present model demonstrated relatively stable performance despite relying solely on NCCT imaging. Similarly, Karamchandani et al. (2022) achieved strong sensitivity using advanced perfusion imaging, whereas the current approach achieved competitive results without requiring additional imaging technologies that are often unavailable in low-resource settings(13). These findings suggest that clinically useful stroke detection models can still be developed using routinely available imaging modalities.

The comparison with the senior radiologist demonstrated that the radiologist achieved slightly higher diagnostic performance than the CNN on the independent evaluation subset. The CNN missed two stroke cases, while the radiologist correctly identified all stroke cases. Nevertheless, the difference in diagnostic performance was not statistically significant (p = 0.21). In contrast, the model processed scans in approximately three seconds per case compared with approximately five minutes required for radiologist interpretation. Similar reductions in interpretation time have been reported in other AI-assisted imaging studies(14). This suggests that AI assisted systems may provide valuable support for rapid triage and preliminary interpretation in emergency settings where specialist radiologists are limited.

A major strength of this study was the integration of the CNN into a functional web-based platform and RESTful API. Unlike many experimental AI models that remain confined to research environments, the present system allows healthcare workers to upload scans, obtain automated predictions, and generate reports through a simple browser-based interface. This increases the potential for future real-world implementation in hospitals with limited radiology expertise.

The quality of the annotated dataset likely contributed substantially to the observed performance. Consensus annotation by experienced radiologists, combined with preprocessing methods such as intensity normalization, augmentation, and texture enhancement, improved the model’s ability to detect subtle ischemic changes. In addition, dropout, L2 regularization, and five-fold cross validation were implemented to reduce overfitting and improve model generalizability across validation subsets.

### Limitations

This study had several limitations that should be considered when interpreting the findings. The data were obtained from a single national referral hospital, which may limit the generalisability of the results to other settings with different CT scanner models, or imaging protocols. The analysis was based on retrospectively collected scans, meaning the tool was not tested within live clinical workflows where challenges such as incomplete studies, motion artefacts, or upload delays can occur. Although the web-based interface and API were successfully tested on a local server, they were not deployed for daily clinical use, and structured feedback from end users, including radiologists, radiographers, and emergency physicians, was not gathered. Furthermore, the current version of the system does not incorporate visual explanation features such as highlighted regions of interest, which can improve transparency and clinician confidence in automated results. These factors indicate the need for further work before large-scale implementation.

## Conclusion

This study developed a practical system for detecting early ischemic stroke from non-contrast CT scans in a Ugandan hospital setting. The CNN demonstrated strong diagnostic performance, with results approaching those of a senior radiologist while generating automated predictions within seconds. In addition, the model was integrated into a browser-based platform that can be used by healthcare workers with minimal technical training. Because the system was developed using locally acquired imaging data and designed around routinely available hospital infrastructure, it has potential to support faster stroke diagnosis in resource-limited settings where access to specialist radiologists is constrained.

Future work should focus on multicenter validation using data from regional and district hospitals to assess performance across diverse imaging equipment and clinical environments. Pilot implementation within healthcare facilities could help evaluate the system’s impact on diagnostic timelines and patient outcomes. Integration with existing Picture Archiving and Communication Systems (PACS) and electronic medical record systems may further improve workflow efficiency through automated identification of suspected stroke cases. In addition, incorporating visual interpretability features, such as heatmaps or lesion overlays, could enhance clinician confidence and support wider adoption. However, further validation in real-world clinical settings is required before large-scale deployment.

## Figures and Tables

**Table 1:** Diagnostic performance of the ischemic stroke detection model on the validation dataset.

Metric	Mean (%)	Standard Deviation (±)
Accuracy	90.2	1.8
Sensitivity	91.4	1.7
Specificity	89.0	2.1
AUC (ROC)	91.3	1.7

**Table 2:** Comparison of CNN and radiologist performance on the independent 30-case evaluation dataset.

Evaluator	Sensitivity (%)	Specificity (%)	Missed Cases	Avg. Time/Scan
CNN Model	86.7	88.7	2	~ 3 sec
Radiologist	100.0	95.0	0	~ 5 min
